# Transient Neurologic Deficits Following Intramuscular Epinephrine Administration for the Treatment of Anaphylaxis

**DOI:** 10.7759/cureus.45254

**Published:** 2023-09-14

**Authors:** Curtis S Pacheco, James Riesing, Cody Ashcroft

**Affiliations:** 1 Internal Medicine, Brooke Army Medical Center, San Antonio, USA

**Keywords:** epinephrine side effects, epinephrine adverse effects, epinephrine neurologic complication, transient neurologic deficits, intramuscular epinephrine complications, anaphylaxis treatment complications, anaphylaxis

## Abstract

Anaphylaxis is an acute, potentially life-threatening severe allergic reaction commonly caused by foods, insect stings, and medications. Intramuscular epinephrine is the cornerstone of treatment for anaphylaxis in order to reverse immediate symptoms and prevent progression to life-threatening hemodynamic or respiratory collapse. By nature of its mechanism of action, epinephrine may induce a number of neurovascular-related adverse effects; even at usual therapeutic doses. Rarely described adverse events include transient ischemic attacks, ischemic stroke, intracerebral hemorrhage, and myocardial infarction. These events may be observed more frequently in patients with cardiovascular risk factors including hypertension, hyperlipidemia, and diabetes mellitus. We present a case of transient neurologic deficits in a patient with underlying cardiovascular disease related to intramuscular epinephrine use for the treatment of anaphylaxis. This case serves to further highlight serious adverse neurologic events that may result from intramuscular epinephrine administration.

## Introduction

Anaphylaxis is a severe, potentially life-threatening allergic reaction that may occur after exposure to foods, insect stings, and medications [1,2]. The preferred and only life-saving treatment for anaphylaxis is epinephrine. Intramuscular (IM) epinephrine injection is recommended due to ease of administration, rapid and reliable rise in plasma/tissue concentration, and lower risk of adverse cardiac effects. Intravenous (IV) epinephrine administration by continuous infusion may be used in cases refractory to IM epinephrine or in intensive care unit settings. Antihistamines, corticosteroids, and bronchodilators are commonly used as adjunctive treatments for symptomatic relief, but do not reverse the underlying mechanism of anaphylaxis or prevent progression to hemodynamic or respiratory collapse.

Epinephrine is a non-selective alpha-adrenergic and beta-adrenergic agonist that is effective in the treatment of anaphylaxis by causing increased vasoconstriction, increased peripheral resistance, positive chronotropy and inotropy, bronchodilation, and decreased soft tissue edema [3]. Common adverse effects from epinephrine administration include anxiety, palpitations, and headaches with more severe adverse effects being ventricular arrhythmias, angina, or even myocardial infarction; however, these are typically only seen in epinephrine overdose [4]. Although not commonly listed, there are a number of reports of adverse neurologic events associated with the use of IM epinephrine including ischemic stroke and intracranial hemorrhage [5-8]. We present a case of transient neurologic deficits following intramuscular epinephrine administration for the treatment of anaphylaxis.

## Case presentation

A 62-year-old male with a history of two prior transient ischemic attacks (TIAs), hypertension, hyperlipidemia, type II diabetes mellitus, and asthma presented to the emergency department with acute onset of tongue swelling, facial flushing, nausea with emesis, dyspnea, and wheezing that developed while eating dinner at a restaurant. He had no prior history of anaphylaxis and no known medication allergies. Vital signs on initial presentation were notable for hypertension to 161/94 mmHg and tachypnea of 26 breaths per minute. On physical examination, he appeared distressed, with prominent angioedema of his tongue and diffuse expiratory wheezing. Notably, he had no complaints of neurologic symptoms and a normal neurologic exam. Initial laboratory evaluation was notable for a leukocytosis of 11.75 x 10^3^ cells/μL and hyperglycemia of 411 mg/dL.

**Figure 1:**
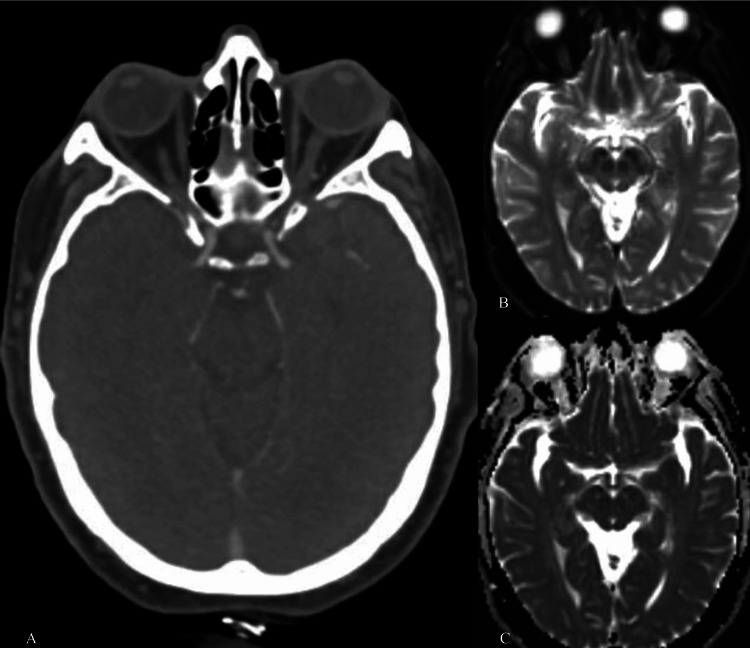
CT Brain, MRI Brain Diffusion-Weighted Imaging, MRI Brain Normal CT brain (A) obtained at the time of the suspected stroke. There is no evidence of bleeding. Additionally, an MRI brain with diffusion weighted (B) and apparent diffusion coefficient (C) imaging was obtained two days later. No evidence of diffusion restriction was identified indicating no ischemic damage. CT: Computed Tomography, MRI: Magnetic Resonance Imaging

He was treated for anaphylaxis to an unknown food allergen with a single dose of 0.5 mg of IM epinephrine in addition to methylprednisolone 125 mg IV, famotidine 20 mg IV, and ipratropium-albuterol 0.5 mg-2.5 mg/3 ml nebulized inhalation. Within five minutes of receiving IM epinephrine, the patient developed dysarthria and right hemiparesis. A Code Stroke was initiated, and a computed tomography (CT) was performed immediately; CT head without contrast (Figure 1A), CT angiogram head, and CT angiogram neck were all unremarkable. Neurology evaluated the patient, and he was found to have an NIHSS of 9: aphasia (+1), left-sided arm drift (+1), right-sided hemiparesis (+6), and right-sided hemisensory deficits (+1). His neurologic findings spontaneously resolved 56 minutes after epinephrine administration. The decision was made by neurology to withhold tenecteplase due to the rapid resolution of symptoms and absence of a causative lesion on imaging. Notably, the patient’s vital signs at the onset of his acute neurologic symptoms and throughout this episode were stable and notable for mild hypertension (152/83-161/70 mmHg) and tachycardia (106-113 beats per minute). He was subsequently admitted for overnight observation. A magnetic resonance imaging (MRI) brain without contrast (Figure 1B-1C) was performed approximately 24 hours after the initial event and showed no evidence of acute infarcts. The patient was then discharged home in his usual state of health. On follow-up evaluation with Allergy/Immunology, no definitive allergen was identified, suggesting either idiopathic anaphylaxis or a toxic reaction to pepper. Ten months later, he has had no further neurologic episodes.

## Discussion

Traditionally, adverse effects associated with epinephrine use are related to overdose of epinephrine, especially in patients with advanced age and predisposing risk factors [4,9]. However, there are several reports of adverse neurologic events associated with IM epinephrine administration at usual therapeutic doses for the treatment of anaphylaxis. These include intracerebral hemorrhage, ischemic stroke, and TIA. In these cases, given the temporal association with IM epinephrine administration and subsequent neurologic deficits, the proposed etiology is acute severe hypertension from the alpha-adrenergic mediated vasoconstrictive effect of epinephrine [5-7]. In our patient, small vessel cerebral vasospasm from the same mechanism is postulated to have caused his predominately unilateral and transient neurologic deficits, which is also supported by the temporal association between IM epinephrine administration and symptom onset. A diagnosis of transient neurologic deficits, secondary to intramuscular epinephrine-induced vasospasm, was made by neurology given the temporal association with epinephrine administration, rapid resolution, and subsequent MRI without evidence of ischemia.

Our patient had multiple risk factors, including type II diabetes mellitus, hypertension, hyperlipidemia, and prior TIA's which are believed to have resulted in increased susceptibility to the alpha-adrenergic agonistic effects of epinephrine. This manifested as transient neurological deficits from a single therapeutic dose of IM epinephrine. A clear association between delayed administration of IM epinephrine and increased mortality in patients experiencing anaphylaxis has been established, therefore early treatment with IM epinephrine is widely recommended [1,4,10,11]. In patients with neurovascular and cardiovascular risk factors, the likelihood of adverse events related to IM epinephrine may be increased. However, there are no absolute contraindications to epinephrine administration given there are no alternatives for definitive management of anaphylaxis [12]. In this patient population, providers may consider using the lowest effective dose of epinephrine or attempt to optimize the patient's hemodynamic status before administration. Ultimately, diagnosis and treatment of anaphylaxis is time sensitive, therefore this strategy may be difficult to apply in practice.

## Conclusions

Intramuscular epinephrine is the mainstay of treatment for anaphylaxis to prevent hemodynamic collapse and reverse the effects of vasoactive mediators. Based on its mechanism of action, there are multiple neurovascular complications, as discussed above, that may occur following IM epinephrine administration, the likelihood of which may be increased in those with underlying neurovascular and cardiovascular disease. All providers who treat anaphylaxis should be aware of these potential adverse events and be prepared to manage them with rapid neurologic and cardiac evaluation, and if appropriate, with IV thrombolytics. This case serves to further illustrate one of the many potential adverse neurological events associated with IM epinephrine at usual therapeutic doses, which may be observed more frequently in patients with underlying neurovascular and/or cardiovascular disease.
